# Antibacterial Activity of Chalcone and Dihydrochalcone Compounds from* Uvaria chamae* Roots against Multidrug-Resistant Bacteria

**DOI:** 10.1155/2018/1453173

**Published:** 2018-08-27

**Authors:** H. Koudokpon, N. Armstrong, T. V. Dougnon, L. Fah, E. Hounsa, H. S. Bankolé, F. Loko, E. Chabrière, J. M. Rolain

**Affiliations:** ^1^Research Unit in Applied Microbiology and Pharmacology of natural substances, Research Laboratory in Applied Biology, Polytechnic School of Abomey-Calavi, University of Abomey-Calavi, 01 P.O. Box 2009, Cotonou, Benin; ^2^Aix Marseille Université, IRD, APHM, MEPHI, IHU Méditerranée Infection, 19-21, Boulevard Jean Moulin, 13385 Marseille Cedex 5, France

## Abstract

This study presents antimicrobial properties of* Uvaria chamae* roots, commonly used for the treatment of various infections in south Benin. Their constituents were extracted and then fractionated in order to isolate the active ingredients. Antimicrobial susceptibility tests were performed against several multidrug-resistant bacteria using the Mueller Hilton well agar diffusion method. Results showed that ethanol extracts were highly active against Gram-positive* cocci*. This activity was more extensive than that measured from conventional broad-spectrum antibiotics. Indeed, vancomycin-resistant* enterococcus* (VRE) and methicillin-resistant* Staphylococcus aureus* (MRSA) strains were all sensitive to this root extract. The aim of this study was to link the antimicrobial activity of the root to chemical structures. The ion mobility mass spectrometry analysis revealed for the first time the presence of ten chalcone and dihydrochalcone structures responsible for the antimicrobial activity of* Uvaria chamae* ethanol extracts. Two structures were described here for the first time in these roots. These findings confirm and justify the medical properties of these roots used as a traditional medicine.

## 1. Introduction

*Uvaria chamae*, also known as finger root or bush banana, could take its name from the Greek word* chamai* meaning “on the ground”. This plant is originated from tropical forests in the west and centre of Africa. It grows in dry and humid shrub land [1, 2]. Its fruit is edible and its roots show great interest throughout the world. Many activities have been assigned to this plant so far: antiparasitic [3, 4], antiplasmodial [5, 6], antidiabetic [7, 8], anti-diarrheal [9], antifungal [10, 11], anti-inflammatory [12], or antimalarial [13, 14].

In Benin,* Uvaria chamae* roots are sold as antidiabetic [15] and for the treatment of infections [16]. These roots are also often prepared in alcohol as a festive drink or used to heal diseases in villages. Many studies have also demonstrated* in vivo* antimicrobial activities in the case of* Uvaria chamae* roots with reference strains [17–19] and multidrug-resistant strains [20]. Yet, no study has identified the compounds responsible for this antimicrobial activity. This present study now addresses the* in vitro* antimicrobial activity of* Uvaria chamae* root extracts and reveals the related compounds.

## 2. Materials and Methods

### 2.1. Root Collection and Extraction

*Uvaria chamae* roots were collected in the district of Cocotomey (Abomey-Calavi, Bénin). Samples were stored at room temperature before further extraction. They were then rinsed and dried at room temperature (16°C) for two weeks and then grinded in a mill. The resulting powder was then extracted in water, ethanol (VWR, Fontenay-sous-Bois, France), and water/ethanol according to a standard protocol published earlier [15]. Water extracts were prepared as follows: 25 g of dry powder was dissolved in 500 mL of distilled water and then heated at 100°C during 30 min. Alcohol extracts were prepared as follows: 25 g of powder was dissolved in 500 mL of ethanol or a 50% ethanol/water mixture (50/50, v:v). All extracts were then filtered through filter paper (VWR) and lyophilized (Cosmos, Cryotec, Saint-Gély-du-Fesc, France). Alcohol extracts were first evaporated using a rotary evaporator (Stuart gamme RE400, Jeulin, Evreux, France) before lyophilization. Final dry extracts were stored at 8°C.

### 2.2. Direct Antimicrobial Activity Determination of Root Extracts

Antimicrobial activity was evaluated using Mueller-Hinton agar culture plates and the agar well diffusion method [21]. All tested strains were first suspended and seeded according to the French Society of Microbiology Antibiogram Comity guidelines [22].

Two strains were originated from the ATCC collection (Teddington, UK). All the other strains were clinical multidrug-resistant isolates (Meti R, Mupi R, Van A/B, BLSE, Vim-2) collected from the public hospital (IHU Mediterranee Infection, La Timone, APHM, Marseille France). Plant extracts were prepared at 50 mg/mL in DMSO (Sigma-Aldrich, Saint-Louis, USA) and then filtered using 0.4 *μ*m multipore membranes (Millex, Merck Millipore, Darmstadt, Germany) in order to be sterile. 50 *μ*l of each plant extract was placed in 6 mm wells dug in the agar plates as previously described [23]. DMSO was also tested as a negative control. Positive controls were conventional antibiotic test discs (SIRscan, Montpellier, France) including vancomycin and mupirocin for Gram-positive* cocci* and imipenem and colistin for Gram-negative* bacilli*. Culture plates were left at room temperature for one hour for prediffusion and then incubated at 37°C during 18 hours as described previously [13]. Each activity test was performed three times to ensure results consistency. Culture inhibition diameters were measured and compared to normalized values previously described [24]. The average of each inhibition diameter obtained was compared with the mean diameters obtained for reference antibiotic discs (Vancomycin and Mupirocin for* Staphylococcus *spp., Vancomycin for* Enterococcus *spp., and Colistin and Imipenem for Negative Gram bacilli strains). Graph Pad software version 6.00 at the significance level of 5% has been used.

### 2.3. MIC, MBC, and Antibacterial Activity Measurements

The Minimum Inhibitory Concentration (MIC) was measured according to a previously reported method performed in microwells with several lines per plate [25]. Each culture plate (Thermo Fisher, California, United States) included 8 lines. Each line included 12 wells. Line 1 was the positive control: bacterial suspension in Mueller-Hinton broth. Line 8 was the negative control: DMSO in Mueller-Hinton broth. Lines 2 to 7 were dedicated to different bacteria strains tested as follows. Well 1 contained 180 *μ*L of 5 mg/mL plant extract. Wells number 2 to 10 were filled with 180 *μ*L of plant extract successively diluted into Mueller-Hinton broth (two per two). Wells 11 and 12 were filled with 180 *μ*L of Mueller-Hinton broth.

Wells 1 to 11 were spiked with 20 *μ*L of Mueller-Hinton broth containing 10% of bacteria measured at 0.5 McFarland. Well 12 did not contain any bacteria in order to check broth sterility. Culture plates were then agitated during 5 minutes and incubated at 37°C for 18 hours. Each well received 40 *μ*L of 0.2% p-iodonitrotetrazolium (INT) aqueous solution (Sigma-Aldrich, Missouri, United States). Plates were stored 20 minutes and protected from light. Red colored wells indicated the viability of bacteria as described previously [26]. Thus, MIC was defined as the lowest concentration corresponding to viable bacteria. Wells without red color were cultivated on new Muller-Hinton culture plates. Minimum Bactericidal Concentration (MBC) was defined as the lowest concentration corresponding to the presence of colonies after culture. Antibacterial power (AP) was defined as the MBC/MIC ratio.

### 2.4. Chemical Properties of Active Compounds

Several tests were performed in order to learn about the chemical nature of the active compounds. Root extracts were heated at 90°C for 30 minutes and reconstituted if necessary. Concentrated extracts were diluted in water at weak and strong pH values (pH 2 and 10 adjusted with NaOH or HCl). Finally, enzymatic digestion was performed on aqueous dilutions using 100 *μ*g/ml of proteinase K (Sigma-Aldrich) at 50°C in 50 mM Tris-HCl and 5 mM CaCl2 (pH 7.5).

### 2.5. Isolation of Active Compounds

100 *μ*l of concentrated ethanol extract (100 mg/mL) was first evaporated under a stream of nitrogen at 40°C. The chemicals were then dissolved in 100 *μ*l of water at pH 10 and the solution centrifuged at 16000* g*. The clear supernatant was collected, leaving behind a pellet of insoluble molecules. 10 *μ*l of supernatant was injected on a UHPLC-UV (Ultrahigh Performance Liquid Chromatography–Ultraviolet) chromatography system in order to separate the compounds (Acquity H-Class, Waters, Saint Quentin en Yvelines, France).

Molecules were eluted at 0.5 mL/min through a phenyl-hexyl column (BEH 1.7 *μ*m 2.1 x 50 mm, Waters) using the following ULC/MS-grade solvents and reagents (Biosolve, Dieuze, France): A, pH 10 aqueous buffer made of 100 mM acetic acid and 100 mM triethylamine; B, methanol. Solvents binary composition consisted in a linear concentration gradient from 10 to 90% of B during 7 minutes. Photodiode array UV detection was set from 200 to 800 nm. Fractions were collected in polypropylene tubes every 30 seconds in order to thereafter check antimicrobial activity.

### 2.6. Active Compounds Identification

The fractions found to be active against selected bacteria were then pooled for subsequent LC/MS analysis using an Acquity I-Class chromatography system connected to a Vion IMS QTOF ion mobility high resolution Quadrupole-time-of-flight mass spectrometer (Waters). Active compounds were separated in the same conditions as for fraction collection. Chemicals were ionized using an electrospray source in the Sensitivity negative mode set as follows: capillary voltage 2.5 kV, source/desolvation temperatures 120/250°C. Ions were monitored using a HDMSe data independent analysis method between 50 and 1000 m/z as follows: scan time 0.1 s, collision energy ramp 20-40 eV, automatic mass correction during survey (using lockmass 554.2620 m/z from a Leucine Enkephalin solution). All ions produced in-source were alternatively analysed at low/high fragmentation energy in order to collect parent/fragments masses throughout the analysis. Raw data was then processed in order to collect ion components defined by a retention time, drift time in the ion mobility cell, parent, and fragments times of flight. Following ion mobility cell and time of flight tube calibrations, the software was capable of calculating Collision Cross Section (CCS) and mass-to-charge values for all generated ions.

The most intense chromatographic signals were then elucidated using the Discovery tool in the UNIFI software (version 1.8, Waters) as follows: automatic elemental composition with 5 mDa tolerance on parent mass, Chemspider as the structure database, and automatic fragment match with 0.5 mDa delta. Structure matches were only considered if the isotope envelope i-fit score was above 80% with at least 1 fragment match. Likely structures were then saved as an in-house database and raw MS data was then processed in order to properly identify suspected structures. Filters were applied on results as follows to confirm identified components: [M-H]^−^adducts only, parent mass error < 1 ppm, at least 3 fragments within 10 mDa tolerance, parent isotopes masses error < 10 ppm (root mean square), and parent isotopes intensities deviation < 20% (root mean square). Experimental fragmentation peaks were attributed to structures using the UNIFI mass fragment prediction tool. Concerning structure isomers with same chemical formulae (same exact masses), the structure attribution was addressed according to the measured Collision Cross Section (CCS) values. Indeed, CCS values increase with the steric crowding of chemicals (structures showed in Figures 2 and 3).

## 3. Results

### 3.1. Antibacterial Activity

First of all, culture inhibition from* Uvaria chamae* root extracts was only observed for Gram-positive* cocci* including the following tested strains* Staphylococcus aureus*,* Enterococcus faecalis*, and* Enterococcus faecium* (Table 1). The inhibition diameters were greater with ethanol extracts than with water-ethanol ones. No activity was observed with water extracts (neutral pH). The highest activity found in ethanol extracts is attributable to higher extraction efficiency with this solvent. Moreover,* Uvaria chamae* ethanol extracts showed high inhibition diameters and presented equivalent inhibition for all tested Gram-positive bacteria including multidrug-resistant strains. For instance,* S. aureus* resistant to mupirocin (mupi R) was not sensitive to mupirocin but was sensitive to the ethanol extract.

Methicillin-resistant* S. aureus *(MRSA or meti R) was also sensitive to the ethanol extract. Similarly,* E. faecalis* and* faecium* both vancomycin-resistant enterococcus (VRE or Van A/B) were also sensitive to ethanol extracts. On the other hand, no inhibition was observed for Gram-negative strains, except with colistin/imipenem control discs.

### 3.2. MIC, MBC, and Antibacterial Activity Calculations

The MIC, MBC, and antibacterial activity calculations on the ethanol extract confirmed an antimicrobial capacity on* Enterococcus* and* Staphylococcus* strains (Table 2). These low *µ*g/mL values for the plant extract indicate an interesting antibacterial power that should be lower if calculated with the pure compounds.

### 3.3. Chemical Properties

**Figure 1:**
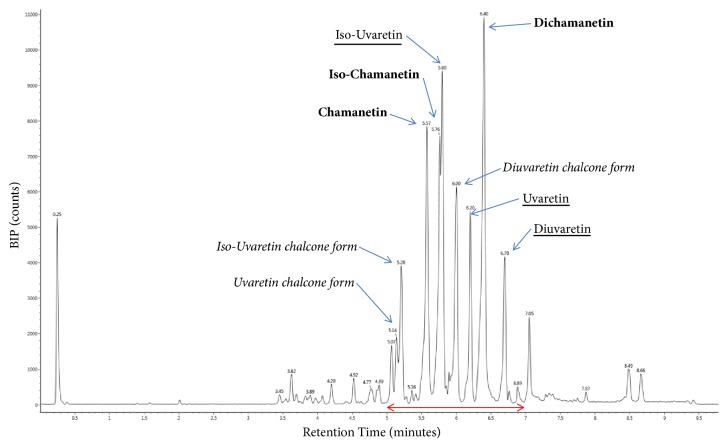
Base Peak Intensity (BPI) extracted LC/MS chromatogram of the pooled active fractions. Identified peaks are labelled and the red line indicates the antimicrobial activity.

First tests indicated that the activity of extracts was maintained after heating, protein digestion and adjustment towards alkaline pH values. Consequently, active compounds were not proteins. According to the solubility of the extracted active compounds in water adjusted at high alkaline pH (>9), one could suppose that their structures contain basic hydroxyl functional groups. Furthermore, high chromatographic retentions using a phenyl-hexyl column suggested structures with aromatic rings. Moreover, the color of the solution and UV absorption (325-330-339 nm maximums) during chromatography analysis also suggested the presence of conjugated unsaturations on these structures. UV absorption maximum suggested 3 types of chromophores. Chromatograms showed several intense peaks at the end of the elution gradient (Figure 1), corresponding to antimicrobial active retention times between 5 and 7 minutes (four fractions of 30 seconds). Each active individual fraction contained at least one intense peak and showed similar antimicrobial activity (data not shown). Chromatograms presented identical retention profiles between UHPLC-UV used for fractionation and UHPLC-MS dedicated to the identification of structures.

### 3.4. LC/MS Identifications

**Figure 2:**
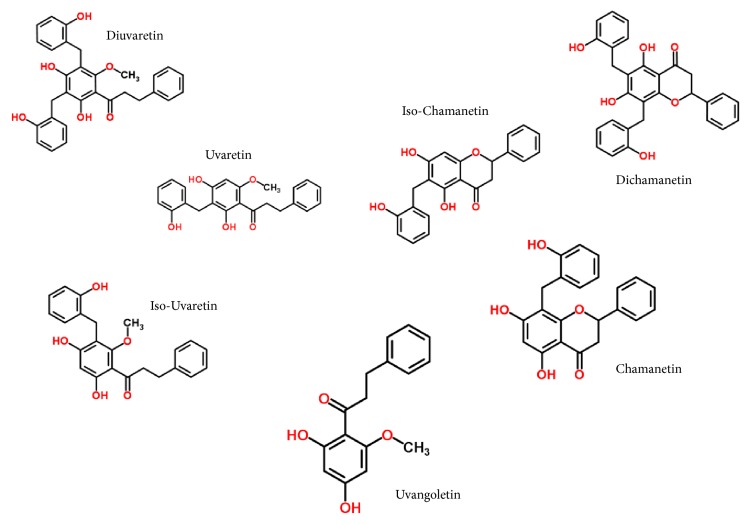
Dihydrochalcone structures identified from the active fractions.

**Figure 3:**
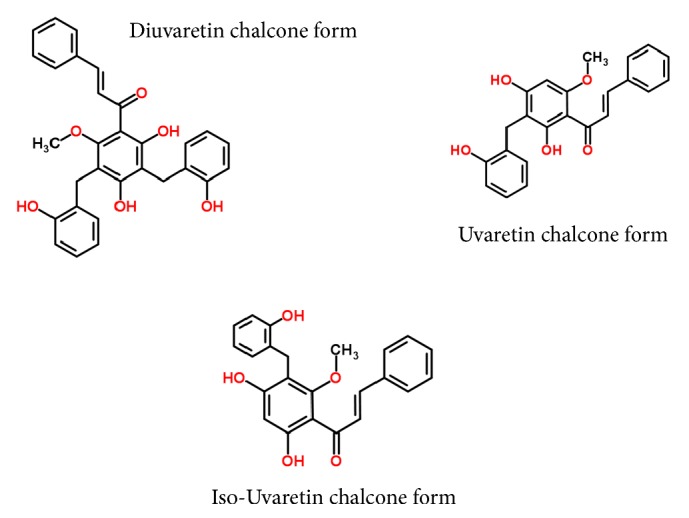
New chalcone structures identified from the active fractions.

**Figure 4:**
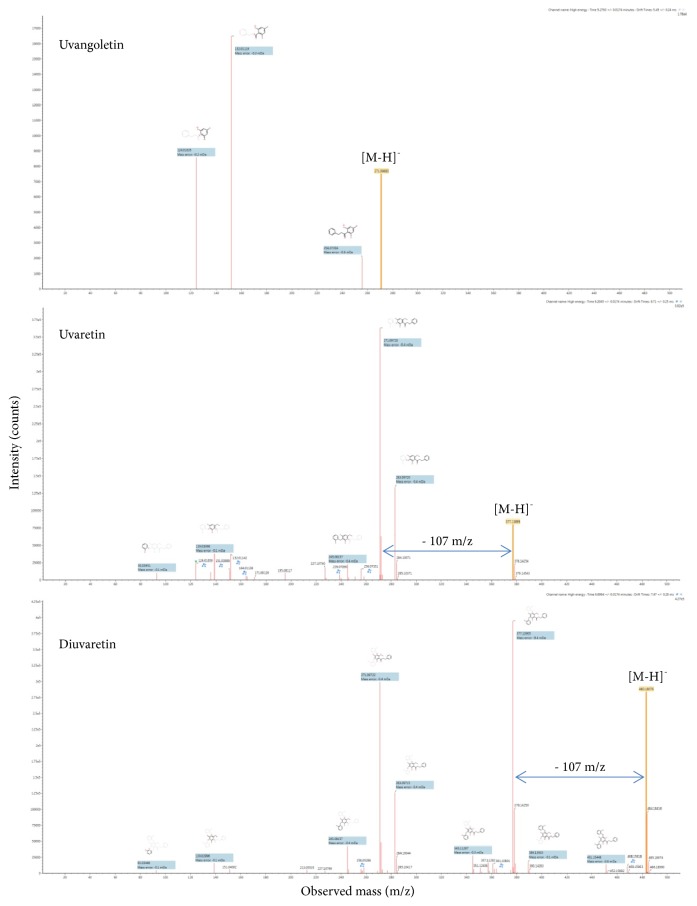
CID fragmentation mass spectra of identified Diuvaretin Uvaretin and Uvangoletin dihydrochalcone structures. Parent peaks and matched predicted structure fragments are labelled. Hydrobenzyl losses (- 107 m/z) are marked.

High-definition LC-MS analysis of the pooled active collected fractions revealed the presence of flavonoid compounds that correspond to the supposed chemical functions (Table 3). Structure elucidation of the most intense peaks was performed using the corresponding ion mobility extracted mass spectrometry data. Structure elucidation search results against the Chemspider structure database showed the confident identity of known dihydrochalcone compounds from the Uvaretin structure family (including Diuvaretin, Uvaretin, isouvaretin, and Uvangoletin) and the Chamanetin structure family (including Dichamanetin, Chamanetin, and iso-Chamanetin). Compounds identifications were confirmed with parent mass errors below 1 ppm (error according to chemical formula) and at least 3 predicted fragments below 10 mDa error. The corresponding dihydrochalcone structures are detailed in Figure 2. Two dihydrochalcone structures presented two hydrobenzyl position isomers each (same exact mass with different retention times and CCS values), namely, Chamanetin and iso-Chamanetin, as well as Uvaretin and iso-Uvaretin.  Figure 4 presents the experimental collision induced dissociation (CID) fragmentation mass spectra of Diuvaretin, Uvaretin, and Uvangoletin identified structures. The identity of compounds within the same family was confirmed by specific parent mass differences between family members corresponding to one or two hydrobenzyl functions losses (delta 107 m/z). Another interesting identified structure family is defined by an unsaturated dihydrochalcone skeleton (or chalcone form). Several structures are proposed for the first time in these roots and named here “Diuvaretin chalcone”, “Uvaretin chalcone”, and “iso-Uvaretin chalcone”. The “Diuvaretin chalcone” and “isouvaretin chalcone” structures were deposited to the Chemspider and Pubchem databases as identification numbers ID 60596760/CID 132275002 and ID 65791003/CID 134686676, respectively. Latest chalcone structures are detailed in Figure 3.

They present the same skeleton structures as Uvaretin family compounds containing one unsaturation (minus two hydrogenes), probably a double bond on the chalcone chain. The presence of an additional double bond compared to dihydrochalcones could induce a stereochemistry site and limits the flexibility of the structure. This could explain the loss of chromatographic retention for these latest structures because of less interaction with the phenyl bonding. Furthermore, these chalcone structures show very close CCS values to their corresponding dihydrochalcone structures. Besides, ion mobility only reveals the presence of a single protomer peaks, except for Uvaretin. It seems therefore that the chalcone structures present their double bond in a single configuration (either E or Z). Finally, all the identifications were consistent with the experimental retention times and drift times. Indeed, structures with larger chemical formula and more important steric crowding (for isomers) showed higher chromatographic retentions and CCS values. For instance, Diuvaretin was the last eluted structure (CCS value = 213.6 Å^2^), whereas Uvaretin, which is a smaller structure lacking one hydrobenzyl function, eluted earlier and showed a smaller CCS value (195.5 Å^2^).

## 4. Discussion

The aim here was to describe the antimicrobial activity of* U. chamae* roots and link this activity to chemical compounds. First, the evaluation of the antimicrobial activity of* Uvaria chamae* root extracts indicated high activity for ethanol extracts according to previously described standard diffusion methods [24, 27]. This activity was only measured against Gram-positive* cocci* and none was visualized against any of the tested Gram-negative strains. A previous study presented similar results [20], that is, no activity of* Uvaria chamae* root ethanol extract against* Klebsiella pneumoniae* a Gram-negative strain, but a strong activity against MRSA strains (methicillin-resistant* Staphylococcus aureus*) a Gram-positive strain.

Nowadays, vancomycin or mupirocin antibiotics are often the last treatment solution against multidrug-resistant bacteria. In comparison with these latest antibiotics,* Uvaria chamae* ethanol root extracts showed here a more extensive inhibition activity, therefore indicating a good potential as an efficient antibiotic.

LC/MS analyses helped to identify the active compounds with confidence. Indeed, the association of chromatographic and ion mobility separations was necessary to extract accurately the associated high resolution mass spectrometry signals of the most intense individual compounds. Mass spectrometry data indicated that the microbial activity measured in collected fractions was linked to seven dihydrochalcone structures (namely, Diuvaretin, Dichamanetin, Uvaretin, Iso-chamanetin, Isouvaretin, and Uvangoletin) and three chalcone structures (namely, “Diuvaretin chalcone form”, “Uvaretin chalcone”, and “isoUvaretin chalcone”). The identification of several similar chemical structures consolidates here the identification of these suspected structures. All structures are part of the flavonoids class within the polyketide lipid category (Lipid MAPS classification). All of them have a common carbon backbone, with varying numbers of hydrobenzyl functional groups that could explain the similar activity in multiple tested fractions. Dihydrochalcone structures were well described previously [28] and are all associated with* Uvaria chamae* according to the literature. Several studies already spotted the implication of chalcones [29–31] and dihydrochalcones [32, 33] as antimicrobial chemicals.

As presented here, [34] also found a strong activity of chalcones against Gram-positive cocci (*Staphylococcus aureus* and* Streptococcus pyogenes*) compared to Gram-negative bacteria (*Salmonella typhimurium* and* Escherichia coli*). Antimicrobial activity of dihydrochalcones form* Populus balsamifera* tree buds was also reported before [35]. The different identified compounds therefore correspond to antimicrobial properties detailed so far in the literature but were not yet associated with* Uvaria chamae* roots as antibiotics. Furthermore, the three chalcone structures described here had never been associated with* U. Chamae *neither.

Two suspected structures were not present in databases and were deposited in Chemspider and Pubchem. These results therefore confirm the advantage of using such root extracts in traditional medicine to heal various diseases.

Concerning the medical use of such compounds, few studies existed so far and were not related to antibiotic treatments. So far, chalcones and dihydrochalcones only showed medical treatment potential as anticancer agents [36–38] or skin protection ingredients [39]. Clinical trials lately demonstrated that several chalcone compounds were not toxic and well tolerated by humans [40]. Dihydrochalcone compound named “hesperidin dihydrochalcone” is nowadays used as a flavour enhancer and does not present any toxicity. Dihydrochalcone and chalcone structures reveal nowadays interesting perspectives in various clinical fields.

## 5. Conclusion

This study addresses the strong antibacterial activity of ethanol extracts from* Uvaria chamae* roots, in particular against Gram-positive multidrug-resistant species. This activity is endorsed by the confident and consistent ion mobility-mass spectrometry identification of chalcone and dihydrochalcone flavonoid structures, beforehand isolated from the plant roots. This study is the first to associate these flavonoid chemicals with this root in the case of an antimicrobial treatment. Therefore, three chalcone structures were suggested for the first time as constituents of* Uvaria chamae* roots. Taken together, these new findings affirm that traditional medicine using this root is of great value for the treatment of microbial infections. This knowledge about flavonoid active ingredients will be helpful for future medication and research in this area. Furthermore, such chalcone and dihydrochalcone compounds show a great potential in the challenging treatment of multidrug-resistant bacteria. Finally, further chemical description and antimicrobial activity measurements of each individual detected compounds are important perspectives that should be addressed in the future.

## Figures and Tables

**Table 1:** Inhibition diameters measured for root extracts and antibiotic discs.

Strain name	Reference	*Uvaria chamae *roots			Antibiotic discs (controls)			
		Water Extract	Ethanol-Water Extract	Ethanol Extract	colistin	imipenem	vancomycin	mupirocin
*S. aureus*	ATCC 259223	0	9.0±0.6 a,b	27.7±0.9 a,b	-	-	20.3±0.9	30.7±0.7
*S. aureus* Méti R	Clinical isolate	0	9.0±0.6 a,b	25.0±0.6 a,b	-	-	20.67±0.3	31.3±1.3
*S. aureus* Mupi R	Clinical isolate	0	9.0±0.6 a	28.3±0.3 a	-	-	19.3±0.3	0
*E. faecalis Van A *	Clinical isolate	0	15.7±0.3	30.7±0.7	-	-	0	-
*E. faecium Van A *	Clinical isolate	0	15.7±0.3	30.3 ±0.3	-	-	0	-
*E. faecium Van B *	Clinical isolate	0	15.7±0.3	28.3±0.3	-	-	0	-
*E. coli *	ATCC 25922	0	0	0	20	28	-	-
*E. coli BLSE *	Clinical isolate	0	0	0	20	28	-	-
*K. pneumoniae BLSE *	Clinical isolate	0	0	0	20	28	-	-
*E. cloacae BLSE *	Clinical isolate	0	0	0	20	28	-	-
*P. aeruginosa Vim-2 *	Clinical isolate	0	0	0	20	0	-	-

Inhibition diameters are given in millimetres. Significant inhibition difference with a: vancomycin and b: mupirocin (p< 0,05).

**Table 2:** MIC, MBC, and antibacterial power (AP) for selected ethanol plants extracts.

	*Uvaria chamae* (ethanolic extract)		
	MIC	MBC	AP
*Staphylococcus aureus* ATCC 25923	0,0046	0,0093	1^*∗*^
*Staphylococcus aureus* Méti R	0,0046	0,018	2^*∗*^
*Staphylococcus aureus* Mupi R	0,0046	0,037	2^*∗*^
*Enterococcus faecalis Van A *	0,0023	0,037	4
*Enterococcus faecium Van A *	0,0023	0,075	4
*Enterococcus faecium Van B *	0,0023	0,075	4

^*∗*^Bactericidal power MIC and MBC were expressed in mg/mL of plant.

**Table 3:** Identified compounds within the antimicrobial fractions.

**Retention Time ** **(min) **	***λ* max ** **(nm) **	**Observed m/z ** **[M-H]**^−^	**Identified structures **	**Mass error ** **(ppm) **	**Matched Fragments **	**Observed CCS ** **(Å**^**2**^**) **	**ChemspiderID **
5.14	330	375.1235	“Uvaretin chalcone form” (2E)-1-[2,4-Dihydroxy-3-(2-hydroxybenzyl)-6methoxyphenyl]-3-phenyl-2-propen-1-one	0.1	15	193.6	21375497

5.20	325	375.1237	“iso-Uvaretin chalcone form” (2E)-1-[4,6-Dihydroxy-3-(2-hydroxybenzyl)-2methoxyphenyl]-3-phenyl-2-propen-1-one	- 0.1	12	190.5	65791003^a^

5.28	-	271.0971	Uvangoletin	- 1.0	3	166.5	4984098

5.57	325	361.1082	Chamanetin	0.7	11	184.3	10305898

5.76	330	361.108	iso-Chamanetin	1.0	10	189.3	10305892

5.80	325	377.1393	iso-Uvaretin (Chamuvaritin II)	0.9	21	191.7	133675

5.99	339	481.1653	“Diuvaretin chalcone form” (2E)-1-[2,4-Dihydroxy-3,5-bis(2-hydroxybenzyl)-6methoxyphenyl]-3-phenyl-2-propen-1-one	- 0.2	26	219.1	60596760^a^

6.20	330	377.1391	Uvaretin	0.1	15	195.5	66150

6.40	330	467.1498	Dichamanetin	0.1	14	215.2	9894419

6.70	339	483.1812	Diuvaretin	0.2	21	213.6	2342170

^a^new structure deposited in Chemspider and Pubchem.

## Data Availability

The data used to support the findings of this study are available from the corresponding author upon request.

## References

[1] Arbonnier M. (2009). *Arbres, Arbustes et Lianes des Zones Seches D’Afrique de L’Ouest*.

[2] Bongers F., Parren M. P., Traoré D. (2005). *Forest Climbing Plants of West Africa: Diversity, Ecology, and Management*.

[3] Fall D., Badiane M., Ba D. (2003). Antiparasitic effect of Senegalese Annonaceae used in traditional medicine. *Dakar Médical*.

[4] Adelodun V. O., Elusiyan C. A., Olorunmola F. O. (2013). Evaluation of antitrypanosomal and anti inflammatory activities of selected Nigerian medicinal plants in mice. *African Journal of Traditional, Complementary, and Alternative Medicines: AJTCAM/African Networks on Ethnomedicines*.

[5] Okokon J. E., Ita B. N., Udokpoh A. E. (2006). The in-vivo antimalarial activities of *Uvaria chamae* and *Hippocratea africana*. *Annals of Tropical Medicine and Parasitology*.

[6] Addae-Kyereme J., Croft S. L., Kendrick H., Wright C. W. (2001). Antiplasmodial activities of some Ghanaian plants traditionally used for fever/malaria treatment and of some alkaloids isolated from *Pleiocarpa mutica*; in vivo antimalarial activity of pleiocarpine. *Journal of Ethnopharmacology*.

[7] Emeka E. J., Oluwatoyin A. E., Adekunle O. I., Ignis I. O. (2015). Preliminary phytochemical screening and evaluation of hypoglycemic properties of the root extract of *Uvaria chamae*. *Bangladesh Journal of Pharmacology*.

[8] Olorunnisola O. S., Adetutu A., Owoade A. O., Okoh O. O., Oyewo E. B., Adegbola P. (2016). Ethno-pharmacological and in-vitro anti-diabetic study of some medicinal plants commonly used in Ogbomoso, South Western Nigeria. *Journal of Applied Biosciences*.

[9] Ambe A. S. A., Ouattara D., Tiebre M.-S., Vroh B. T. A., Zirihi G. N. (2015). Diversité des plantes médicinales utilisées dans le traitement traditionnel de la diarrhée sur les marchés d'Abidjan (Côte d'Ivoire). *Journal of Animal and Plant Sciences*.

[10] Okwuosa O. M., Chukwura E. I., Chukwuma G. O. (2012). Phytochemical and antifungal activities of *Uvaria chamae* leaves and roots, *Spondias mombin* leaves and bark and *Combretum racemosum* leaves. *African Journal of Medicine and Medical Sciences*.

[11] Kongstad K. T., Wubshet S. G., Kjellerup L., Winther A.-M. L., Staerk D. (2015). Fungal plasma membrane H^+^-ATPase inhibitory activity of o-hydroxybenzylated flavanones and chalcones from *Uvaria chamae* P. Beauv. *Fitoterapia*.

[12] Omajali J. B., Hussaini S. J., Omale J. (2011). Cytotoxicity and anti-inflammatory studies on Uvaria chamae. *Journal of Pharmacology and Toxicology*.

[13] Oke M. A., Bello A. B., Odebisi M. B., El-Imam A. M. A., Kazeem M. O. (2013). Evaluation of antibacterial efficacy of some alcohol-based Hand sanitizers sold in Ilorin (NorthCentral Nigeria). *Ife Journal of Science - African Journals*.

[14] Kazembe T., Munyarari E., Charumbira I. (2012). Use of traditional herbal medicines to cure Malaria. *Bulletin of Environment, Pharmacology and Life Sciences*.

[15] Fah L., Klotoé J.-R., Dougnon V. (2013). Étude ethnobotanique des plantes utilisées dans le traitement du diabète chez les femmes enceintes à Cotonou et Abomey-Calavi (Bénin). *Journal of Animal and Plant Sciences*.

[16] Koudokpon H., Dougnon T., Bankolé H., Fah L., Hounmanou Y., Loko F. (2017). Enquête ethnobotanique sur les plantes utilisées dans le traitement des infections au sudbénin. *Health Sciences and Diseases*.

[17] Monon K., Abdoulaye T., Karamoko O., Adama C. (2015). Phytochemical composition, antioxidant and antibacterial activities of root of *Uvaria chamae* P. Beauv. (Annonaceae) used in treatment of dysentery in North of Côte d’Ivoire. *International Journal of Pharmacognosy and Phytochemical Research*.

[18] Ogbulie J. N., Ogueke C. C., Nwanebu F. C. (2007). Antibacterial properties of *Uvaria chamae*, *Congronema latifolium*, *Garcinia kola*, *Vemonia amygdalina* and *Aframomium melegueta*. *African Journal of Biotechnology*.

[19] Osuagwu G., Ihenwosu A. (2014). Phytochemical composition and antimicrobial activity of the leaves of *Alchornea cordifolia* (Schum and Thonn), *Sanseviera liberica* (Gerand Labr) and *Uvaria chamae* (P. Beauv). *American Journal of Phytomedicine and Clinical Therapeutics*.

[20] Oluremi B. B., Osungunna M. O., Omafuma O. O. (2010). Comparative assessment of antibacterial activity of Uvaria chamae parts. *African Journal of Microbiology Research*.

[21] Balouiri M., Sadiki M., Ibnsouda S. K. (2016). Methods for *in vitro* evaluating antimicrobial activity: a review. *Journal of Pharmaceutical Analysis*.

[22] CA-SFM (2017). Recommandations EUCAST pour la réalisation de l’Antibiogramme. *Société Française Microbiol*.

[23] Agbankpe A. J., Dougnon T. V., Bankole S. H., Houngbegnonn O., Dah-Nouvlessouno D., Baba-Moussa L. (2016). In vitro antibacterial effects of Crateva adansonii, Vernonia amygdalina and Sesamum radiatum used for the treatment of infectious diarrhoeas in Benin. *Journal of Infectious Diseases & Therapy*.

[24] Tsirinirindravo L., Andrianarisoa B. (2009). Activités antibactériennes de l’extrait des feuilles de Dalechampia clematidifolia (Euphorbiaceae). *International Journal of Biological and Chemical Sciences*.

[25] Lagnika L., Amoussa M., Sanni A. (2014). In vitro antibacterial activity of two medicinal plants used in Bénin to treat microbial infections. *Indian Journal of Science and Technology*.

[26] Eloff J. N. (1998). A sensitive and quick microplate method to determine the minimal inhibitory concentration of plant extracts for bacteria. *Planta Medica*.

[27] Bauer A. W., Kirby W. M., Sherris J. C., Turck M. (1966). Antibiotic susceptibility testing by a standardized single disk method. *American Journal of Clinical Pathology*.

[28] Parmar V. S., Tyagi O. D., Malhotra A., Singh S. K., Bisht K. S., Jain R. (1994). Novel constituents of Uvaria species. *Natural Product Reports*.

[29] Shah S. S., Goswami K. (2013). Synthesis, characterization and anti microbial activity of some novel chalcone compounds having benzyloxymonochloro resacetophenone moiety. *Der Pharma Chemica*.

[30] Patel N. B., Patel M. D. (2017). Synthesis and evaluation of antibacterial and antifungal activities of 4-thiazolidinones and 2-azetidinones derivatives from chalcone. *Medicinal Chemistry Research*.

[31] Oldoni T. L. C., Cabral I. S. R., D'Arce M. A. B. R. (2011). Isolation and analysis of bioactive isoflavonoids and chalcone from a new type of Brazilian propolis. *Separation and Purification Technology*.

[32] Lavoie S., Legault J., Simard F., Chiasson É., Pichette A. (2013). New antibacterial dihydrochalcone derivatives from buds of Populus balsamifera. *Tetrahedron Letters*.

[33] Simard F., Gauthier C., Chiasson É. (2015). Antibacterial balsacones J–M, hydroxycinnamoylated dihydrochalcones from *Populus balsamifera* Buds. *Journal of Natural Products*.

[34] Khan A. S., Asiri M. A. (2017). Green synthesis, characterization and biological evaluation of novel chalcones as anti bacterial agent. *Arabian Journal of Chemistry*.

[35] Chassion E. (2012). *Isolation, Caractérisation et Évaluation du Potentiel Bioactif des Bourgeons de Populus balsamifera*.

[36] Das M., Manna K. (2016). Chalcone scaffold in anticancer armamentarium: a molecular insight. *Journal of Toxicology*.

[37] Jandial D. D., Blair C. A., Zhang S., Krill L. S., Zhang Y.-B., Zi X. (2014). Molecular targeted approaches to cancer therapy and prevention using chalcones. *Current Cancer Drug Targets*.

[38] Wani Z. A., Luqman S. (2017). Chalcone and their derivatives as anticancer agents. *Topics in Anti-Cancer Research*.

[39] Martinez R. M., Pinho-Ribeiro F. A., Steffen V. S. (2016). Topical formulation containing hesperidin methyl chalcone inhibits skin oxidative stress and inflammation induced by ultraviolet B irradiation. *Photochemical & Photobiological Sciences*.

[40] Sahu N. K., Balbhadra S. S., Choudhary J., Kohli D. V. (2012). Exploring pharmacological significance of chalcone scaffold: a review. *Current Medicinal Chemistry*.

