# Erratum to “Antibacterial Activity of Chalcone and Dihydrochalcone Compounds from* Uvaria chamae* Roots against Multidrug-Resistant Bacteria”

**DOI:** 10.1155/2019/5962198

**Published:** 2019-03-03

**Authors:** H. Koudokpon, N. Armstrong, T. V. Dougnon, L. Fah, E. Hounsa, H. S. Bankolé, F. Loko, E. Chabrière, J. M. Rolain

**Affiliations:** ^1^Research Unit in Applied Microbiology and Pharmacology of natural substances, Research Laboratory in Applied Biology, Polytechnic School of Abomey-Calavi, University of Abomey-Calavi, 01 P.O. Box 2009, Cotonou, Benin; ^2^Aix Marseille Université, IRD, APHM, MEPHI, IHU Méditerranée Infection, 19-21, Boulevard Jean Moulin, 13385 Marseille Cedex 5, France

In the article titled “Antibacterial Activity of Chalcone and Dihydrochalcone Compounds from Uvaria chamae Roots against Multidrug-Resistant Bacteria” [1], minor errors were found which happened during the production process. The following corrections should be applied to the article:

- The name of the same compound is written in different ways throughout the text, i.e., isouvaretin, iso-Uvaretin, Isouvaretin, isoUvaretin, and Iso-uvaretin, as well as iso-Chamanetin, Iso-Chamanetin, and Iso-chamanetin. These formats should be standardized to Iso-uvaretin and Iso-chamanetin.
- In the description of Table 1, p< 0, 05 should be corrected into p<0.05.
- In Figure 1, BIP on the vertical axis should be corrected to BPI.
- The sentence “Antimicrobial activity of dihydrochalcones form Populus balsamifera tree buds was also reported before [35]” should be corrected to “Antimicrobial activity of dihydrochalcones from Populus balsamifera tree buds was also reported before [35].”
- In the Conclusion, the last sentence should be updated from “Finally, further chemical description and antimicrobial activity measurements of each individual detected compounds are important perspectives that should be addressed in the future” to “Finally, further chemical description and antimicrobial activity measurements of each individual detected compound are important perspectives that should be addressed in the future.”
